# General practitioners’ needs for support after the suicide of patient: A qualitative study

**DOI:** 10.1080/13814788.2018.1485648

**Published:** 2018-07-27

**Authors:** Danica Rotar Pavlič, Marta Treven, Alem Maksuti, Igor Švab, Onja Grad

**Affiliations:** a Department of Family Medicine, Faculty of Medicine, University of Ljubljana, Ljubljana, Slovenia;; b Institute for Political Management, Ljubljana, Slovenia;; c Slovenian Association for Suicide Prevention, Ljubljana, Slovenia

**Keywords:** General practitioners, patient suicide, professional assistance, qualitative study, Slovenia

## Abstract

**Background:** Most patients that commit suicide consult their GPs before their death. This topic is often surrounded by secrecy and associated with guilt and shame. There is a lack of knowledge about support for GPs after patient suicide.

**Objectives:** To identify the widest range of Slovenian GPs’ problems and needs in connection with patient suicide, and, based on the findings of the study, to prepare ways to assist GPs after patient suicide.

**Methods:** Semi-structured interviews were held with GPs that had experienced a patient’s suicide during their professional career until saturation was reached. The interview guide was piloted. Twenty-two in-depth interviews were carried out between April 2012 and February 2013. Transcripts were coded and thematically analysed using qualitative content analysis.

**Results:** Participating GPs suggested possible forms of support, most frequently individual consultation with a psychologist or a psychiatrist, in person, by phone, or via e-mail. Balint groups, group consultations and various workshops on suicide or depression would be a preferable form of support. Some GPs perceived critical incident review as an attempt to blame them, whereas others saw it as an opportunity for support. A group of peers that could discuss professional dilemmas in which more experienced GPs would help younger GPs would be helpful.

**Conclusion:** Slovenian GPs did not have any formal support system at the time of the research, but they would appreciate such a possibility.

KEY MESSAGES

- When dealing with the suicide of their patients, Slovenian GPs are often left to informal support structures and self-help resources, like family and colleagues.
- The problems and needs identified in connection with patient suicide are a basis for developing professional support to assist Slovenian GPs after patient suicide.

## Introduction

Slovenia is regarded as a high suicide-risk country. The average suicide rate between 2000 and 2014 was 23.8 per 100,000 inhabitants [1]. Over the past few years, preventive activities in Slovenia have been stepped up. A remarkable achievement has been made in the awareness of the general public and strengthening skills for identifying and dealing with suicidal disadvantages in various target groups [2]. Although the impact of suicide on general practitioners (GPs) is less often considered as a topic, in recent years there have been some significant studies in this area [3–7]. In practically all of the studies, the authors determined that most patients that commit suicide consulted their GP shortly before death. Therefore, GPs need to be in a position to recognize the needs of those that have been bereaved by suicide and also need to look after themselves [4]. GPs that mainly work in community health centres are well-positioned to support family members after the suicide of a relative and to navigate problems associated with the grief process [5]. However, a patient suicide is also a stressful event for the GP [4]. Despite the frequency and substantial distress of this phenomenon, GPs are mostly left to cope with complex situations on their own.

Based on previous research, the reactions of GPs are similar to the reactions of psychiatrists and psychologists. They experience guilt and blame, fear of making mistakes, feelings of helplessness, emotional distance, isolation, feeling upset when thinking about the patient, feeling numb, sadness, physical reactions (such as changes in appetite, fatigue, sleeping problems, etc.), personal experiences of loss, their personal style of coping and the management of their own bereavement, identification with patients’ families, questions of personal competency, intellectual distance, and a sense of moral responsibility for the death [4–9]. Fleming has pointed out that patient suicide causes severe distress in even the most experienced GPs, affects GPs’ clinical acumen and well-being, and can even lead to depression and a GPs’ suicide [10]. He also reported that professional support is sometimes poorly accessible. GPs have difficulties admitting a need for this, and he proposed several initiatives for how it should be approached [10].

There is a lack of knowledge about the attitudes of GPs toward professional support. For example, Danish GPs managed the emotional impact of a suicide on their own without seeking support, although some admitted it would be beneficial to have it [11]. In Ireland, 62% of GPs would use support if it were available [12]. In the UK [3], most GPs affected by patient suicide seek informal support from their peers and colleagues. Although most participants assess this method as relatively successful, they have warned of the lack of guidelines for those that may require formal support. In other words, GPs miss institutions and well-established procedures to ensure the availability of formal support after their patients commit suicide [3].

This study explores Slovenian GPs’ understandings of their needs for formal support after patient suicide. The study’s objective is to identify the widest range of GPs’ problems and needs in connection with patient suicide and to explore the views of GPs on this issue. The findings presented in this article are a continuation of efforts to develop national guidelines to assist GPs after patient suicide.

## Methods

### Study design

This qualitative study used semi-structured interviews to explore GPs’ problems and needs in connection with patient suicide, their beliefs, and reflections that could be difficult to share with a group [13,14]. The goal of the research was to use the findings to prepare professional support to assist GPs after suicide of patients.

### Selection of study subjects

Through purposive sampling, participants were recruited from a population of GPs. The following sampling criteria were applied: registered GP, having a practice in Slovenia, and experience of patient’s suicide during their professional career [15]. We preliminarily checked that all of the participants were willing to take part in our study. All of the participants agreed to be interviewed. We managed to put all groups of GPs into the sample: female and male, older and younger, from rural and urban areas, and variously educated in mental health. Purposive sample size was determined by theoretical saturation, which is the point in the data collection process when new data no longer offer additional insights for the research question [15]. The final number of interviews conducted was 22.

### Qualitative methods

In line with the research goals, we decided to conduct interviews to explore GPs’ needs for support [16]. A semi-structured questionnaire was prepared and developed through a literature review (MT, OG, DRP, IŠ). It was additionally redesigned after a pilot interview (MT, DRP). The interviews were conducted by MT, a sixth-year medical student, mostly at the GPs’ practices. MT had individual training on qualitative methodology, which was led by AM and DRP. The interviews were held between April 2012 and February 2013. They lasted 65 min on average (from 37 to 112 min). They were recorded digitally, transcribed verbatim, and anonymized.

### Outcomes and analysis

MT, AM, and DRP conducted a qualitative content analysis [17]. AM and DRP are teachers in qualitative methods at the graduate level. DRP has written book chapters about qualitative research [18,19].

As a tool to facilitate qualitative data analysis, we used the software ATLAS.ti (Scientific Software Development GmbH, Berlin, Germany). Interview transcripts were read, qualitatively coded, reviewed, and labelled. We used inductive content analysis [20,21]. Each interview was analysed by the authors independently and by generating codes for each statement reflecting the GPs’ experiences and needs. Categories were defined in an inductive, iterative reflection process after having established codes to condense observations from the data; categories had not been predetermined. If consensus was not attained, intercoder agreement on differently perceived parts was processed [22].

## Results

Four categories and 146 codes with a total frequency of 1482 were identified. We determined the following categories: (1) need for support, (2) type and benefits of support, (3) availability of professional support and barriers to using it, and (4) suggestions for future organization of support.

### Need for support

Almost all of the interviewees agreed on the necessity of support for GPs after patient suicide. Some have seen this as their own need, whereas some of them expressed the opinion that they do not need support themselves, but other GPs do. The participants described positive effects on both GPs’ wellbeing and GPs’ work. Suicide was sometimes compared with a traffic accident, a failed resuscitation, or a dead child. Support can help GPs recover faster, allow them to continue with immediate work, and learn from experience. Here are two interesting examples:

I would say yes. It is very useful. When you need support, it is nice to get it. And you know, every suicide is different, and patients are different, so you are in a difficult situation again and again. (GP 21)

Just imagine: a traffic accident, a failed resuscitation, or a dead child, these are terrible things! Sometimes it’s useful just to share your experience with somebody. That is where you can find solace, understanding, compassion, and of course professional help. (GP 9)

### Type and benefits of support

In connection with patient suicide, GPs highlighted a range of different reactions, including fear, anger, stress, changes in appetite, fatigue, sleeping problems, tingling, nausea, powerlessness, guilt, distortion, desperation, affliction, defeat, emptiness, agitation, disappointment, irritability, anxiety, and flushed face.

GPs most often talked to their colleagues, either at their workplace or outside it, followed by a nurse or members of the team. Some of them described the type of informal support and benefits they receive from their colleagues (conversation, relief, and/or legitimacy of proper conduct), followed by a nurse or members of the team, and with their family (usually their partner). On rare occasions, GPs talked to the patient’s family about their feelings.

I did everything right, but. (GP 3)

I am not omnipotent. (GP 7)

GPs admitted that it was difficult to seek professional help from a psychiatrist or another specialist in the past. Seeking help still carries a stigma and because of this GPs often utilize self-help methods that are often constructive (playing with children, recreation, autogenic training, breathing exercises, education, etc.) but also sometimes self-destructive (drinking, smoking, overeating, or even suicidal thoughts).

### Availability of professional support and barriers to using it

Although the idea of support was positively accepted, the GPs highlighted some serious concerns about the availability of professional support in the case of different reactions after a patient’s suicide. Some of the GPs interviewed pointed to few existing forms of professional support. They mentioned an anonymous help centre at the national medical chamber and the lack of systematic professional psychiatric assistance for GPs.

Some GPs confirmed that they experienced intense psychological distress after a patient’s suicide, leading them to self-destructive reactions. Our interviewers noted some fundamental barriers that are responsible for problems in providing professional help to GPs. For example, one GP pointed out:

It takes too long before professional support for a GP is provided. (GP 14)

In this case, he drew attention to

The need for immediate availability of professional support. (GP 17)

Another interviewee highlighted some additional social barriers, such as stigma, mistrust, and a culture of blame. Instead of seeking help, GPs are often stuck in narrow frames of conventional expectations of society where searching for help is sometimes characterized as weakness or professional and personal failure. GPs noted that they miss the systematic regulation of this problem, and they offered concrete suggestions that are discussed below.

### Suggestions for future organization of support

The GPs interviewed commented on many possible forms of support, which can be organized in a professional manner or among peers. Suggestions for individual and group sessions were mentioned as well as a critical incident review (CIR).

The most frequently mentioned form of support was individual consultation with a psychologist or a psychiatrist. Support may take place in person, by phone, or via e-mail. Some GPs prefer a psychologist, who, to their opinion, has better communication skills, treats healthy people, and would not trouble a GP with medical questions. Others prefer a psychiatrist, who could offer answers on professional matters and guidelines for future work. There was also a suggestion that a lawyer should also be available.

Balint groups were mentioned, but with some reservations. One must take into account the small community of GPs in small countries such as Slovenia, who mostly know each other, and so social control may be quite substantial. Some GPs said they would join a Balint group only if it were separate from their workplace and if they did not know the other participants. For some GPs, groups would be the most preferable form of support, and others viewed them as a form of support complementing individual consultations. There was a suggestion for various workshops on suicide or depression.

GPs mentioned that CIR is an attempt to blame them, and others did not see any benefits that it could provide. They considered it an additional burden on them. Some were ambivalent, whereas others saw it as an opportunity for learning, gaining greater professional competence, and at the same time support for GPs. Several GPs suggested that CIR should be optional.

Other forms of support mentioned by GPs were workshops at educational meetings with sufficient time for informal discussion, groups of peers that would discuss professional dilemmas, and meetings with more experienced GPs that could help younger GPs. All of the GPs interviewed agreed that regular meetings of colleagues and ad hoc discussions immediately after a stressful event at the health centre would be helpful. Capacity-building and ongoing medical education on topics such as distress after a patient suicide was mentioned.

## Discussion

### Main findings

The main finding of our study is that Slovenian GPs most often seek support among colleagues and only in exceptional cases resort to professional support. The Slovenian GPs interviewed expressed the need for and interest in professional support much more often than professional support was available. They also offered some suggestions for what should be done to achieve better availability. Another significant finding is that Slovenian GPs blame culture (i.e. social habits) as an important barrier to the use of professional support. Some Slovenian GPs believe that professional support is beneficial in general, but they were not convinced about actually using it. This discrepancy is odd, but we also know that most bereaved relatives do not seek professional support.

### Limitations

The general limitation is related to epistemological criteria and validity in qualitative research [13]. For a more comprehensive picture, additional GPs, psychiatrists and other suicide professionals, and policymakers should be included in the research to provide additional insight.

### Comparison with previous literature

Among the participants that said that they had needed professional support and would have used it, but it had not been available, were the GPs that expressed intense psychological distress after their patient’s suicide (including the use of alcohol and thoughts about their own suicide). These results confirm Fleming’s report that consequences of a patient’s suicide can be intense and deep [10]. Collegial support, which was found in our study, was also found in a Danish study [11]. Comparable results were found among psychiatrists, in which 85.9% of them sought support from colleagues, followed by a supervisor or psychotherapist [23]. A perception of a blaming culture was mentioned in research by Kendall et al. [9]. No articles reporting on the availability of support were found, which might imply a similar lack of support in other countries.

### Implications

Based on our results, we suggest better education programs about the problem of suicide. It would make sense to raise GPs’ awareness of suicide’s social dimension [24]. Training and developing specific post-suicide protocols was suggested by frontline staff in Ireland [25]. Team meetings were commonly held and found to be helpful. A CRI was viewed as helpful by consultant psychiatrists from Scotland [26]. A structure that would formally assist GPs after patient suicide and specific post-suicide protocols is necessary. In Slovenia, the findings of the research have contributed to the establishment of a professional group for doctors and dentists that are in distress at the Medical Chamber of Slovenia.

## Conclusion

This study demonstrates the importance of professional support for GPs after the suicide of a patient. The findings presented in this article represent a realistic framework for when and how to provide adequate and useful professional support to GPs.
